# Suicidality and divalproex sodium: analysis of controlled studies in multiple indications

**DOI:** 10.1186/1744-859X-10-1

**Published:** 2011-01-18

**Authors:** Laura Redden, Yili Pritchett, Weining Robieson, Xenia Kovacs, Mary Garofalo, Katherine Tracy, Mario Saltarelli

**Affiliations:** 1 Abbott, Abbott Park, IL, USA

## Abstract

**Background:**

Recent analyses of antiepileptic drugs have indicated an increase in the risk of suicidality. The objective of this report was to provide clinical information and an independent meta-analysis of divalproex sodium and suicidality events by analyzing data from 13 placebo-controlled studies and 1 low-dose controlled study.

**Methods:**

Adverse events considered to be possibly suicide related were identified using the Columbia Classification Algorithm of Suicide Assessment (C-CASA) methodology. Indications included epilepsy, bipolar disorder, migraine prophylaxis, impulsive aggression, and dementia. Narratives were produced for every event, and suicidality event ratings were performed by a third party blinded to treatment assignment. Statistical analyses were conducted using methodology similar to that reported by the US Food and Drug Administration (FDA).

**Results:**

Suicidality events were identified in 5 of the 13 placebo-controlled studies. Of the 1,327 (0.83%) subjects taking divalproex sodium, 11 had suicidality events: 2 suicide attempts and 9 suicidal ideation. Of 992 (0.91%) subjects taking placebo, 9 had suicidality events: 1 preparatory act toward suicide, 2 suicide attempts, and 6 suicidal ideation. Across placebo-controlled studies, the overall estimated odds ratio (OR) of suicidal behavior or ideation was 0.72 (95% CI 0.29 to 1.84). The OR for suicidal behavior was 0.37 (95% CI 0.04 to 2.58), and the OR for suicidal ideation was 0.90 (95% CI 0.31 to 2.79).

**Conclusions:**

In this meta-analysis, divalproex sodium does not appear to increase the risk of suicide-related adverse events relative to placebo in the populations studied. Clinicians should nonetheless remain vigilant in assessing suicidality, not only in patients treated for mental disorders with inherently high suicide risk, but also in patients taking antiepileptic medications.

## Background

The latest World Health Organization statistics revealed that approximately 800,000 people commit suicide annually worldwide [1]. In the US, the suicide rate was 10.9 per 100,000 and was the second leading cause of death in the 25-34-year-old age group in 2006 [2]. The term suicidality encompasses a spectrum of events of varied severity, ranging from suicidal ideation to suicidal behavior and suicide. Approximately 6 years ago, the US Food and Drug Administration (FDA) evaluated the association between antidepressant agents and the increased risk of suicidality. More recently, this investigation was extended to the use of other medications including antiepileptic drugs (AEDs). FDA analyses employed a retrospective systematic search and adjudication of spontaneously reported possibly suicide-related adverse events from controlled clinical studies [3]. In the case of antidepressants, the results of such analyses led to the addition of a warning to prescribing information regarding increased suicidality risk in the pediatric population. The FDA published their statistical review and evaluation of AEDs and suicidality in 2008 [4], which has also led to prescribing information modifications [5].

Certain patient populations treated with AEDs, such as those with epilepsy and bipolar disorder, are known to be at increased risk of suicide. Evaluating the association between AED therapy and suicidality in these populations may therefore be confounded by the high incidence of suicidality associated not only with disease states *per se*, but the risk associated with comorbid psychiatric conditions. Epilepsy is a disorder associated with considerable affective symptomatology in those with this illness [6-9]. Patients with epilepsy have been reported to be five times more likely to commit suicide than the general population [10]. In addition, 25% of epilepsy patients in the community are thought to experience suicidal ideation compared to 13.3% of patients without epilepsy [11]. A meta-analysis of suicide risk indicated that patients with bipolar disorder are 16 times more likely to commit suicide than the general population [10]. In the most recent Centers for Disease Control Surveillance for Violent Deaths, 13.4% of people who had committed suicide had a diagnosis of bipolar disorder [2]. Among patients admitted to an emergency room for suicide attempts, those attempting suicide were five times more likely to have bipolar disorder than those presenting to the emergency room for non-suicide related psychiatric issues [12].

Divalproex sodium (DVPX) is an AED widely used in epilepsy, the treatment of manic episodes associated with bipolar disorder, and migraine prophylaxis [13]. From 2005 to 2007, the FDA acquired placebo-controlled clinical study data from the manufacturers of 11 different AEDs. The purpose of the meta-analysis was to determine whether the use of AEDs conferred a risk of suicide-related adverse events, and the detailed methods have been presented elsewhere [4]. The primary endpoint of the FDA analysis was suicidal behavior or ideation. Patients with completed suicides, suicide attempts, preparatory acts toward imminent suicidal behavior, or suicidal ideation were considered to meet the primary endpoint. Suicidal behavior (completed suicide, suicide attempt, or preparatory acts toward imminent suicidal behavior) and suicidal ideation were the two secondary endpoints. Subgroup analyses were conducted in each AED individually, as well as by drug group (sodium channel blockers, γ-aminobutyric acid (GABA)ergic and GABAmimetics, carbonic anhydrase inhibitors), trial indication (epilepsy, psychiatric, other), demographic characteristics, setting (inpatient or inpatient/outpatient combined, outpatient), and location (North America, non-North America) [4].

DVPX was among the 11 AEDs assessed by the FDA to determine the potential risk of suicidality from the use of these drugs. A dataset was provided by the sponsor (Abbott, Abbott Park, IL, USA) to the FDA, containing data from 14 clinical trials conducted to evaluate the efficacy and safety of DVPX in various indications. The FDA suicidality meta-analysis of the 11 AEDs included a total of 199 placebo-controlled clinical studies (43,892 subjects) and 11 low-dose-controlled studies (1,587 subjects). In the FDA meta-analysis of placebo-controlled trials, the overall estimated odds ratio (OR) for a suicidal behavior or ideation event was 1.80 (95% CI 1.24 to 2.66) for the combined 11 AEDs when compared to placebo. Individually, DVPX had an OR for a suicidal behavior or ideation event of 0.72 (95% CI 0.29 to 1.84) when compared to placebo. When analyzed by indication, the FDA reported that the OR for a suicidal behavior or ideation event in patients with epilepsy was 3.53 (95% CI 1.28 to 12.10) and was 1.51 (95% CI 0.95 to 2.45) in the psychiatric population [4].

The DVPX prescribing information has been modified to highlight the increased risk of suicidal thoughts and behavior based on the FDA meta-analysis. Since the FDA released their findings, clinicians using AEDs have sought to put the information into a clinical context [14-17]. The objective of this study was to assist clinicians by further contributing to the body of available knowledge regarding suicidality and adverse events, focusing specifically on DVPX. To accomplish this, the same DVPX dataset provided to the FDA was analyzed separately from the FDA meta-analysis. The data summarized in this report are an individualized depiction of suicidality and DVPX, and are distinct from the aforementioned meta-analysis of 11 AEDs. The studies in the following DVPX meta-analysis encompass a broad range of indications including epilepsy, acute mania in bipolar disorder, bipolar depression, dementia, migraine, and impulsive aggression. In addition to overall risk, the risks of suicide-related events were calculated by study and by indication. Details of each suicidality event from the dataset are also presented for the first time.

## Methods

The methods used for the selection of studies, search for possibly suicide-related events and characterization of identified events were based on instructions communicated by the FDA directly to the sponsor. Analyses in this report used the same dataset submitted to the FDA for inclusion in their meta-analysis. A total of 13 placebo-controlled studies and 1 study using a subtherapeutic dose of DVPX as a control were identified; all were sponsored by Abbott (Table 1). Study durations ranged from 3 to 52 weeks, with a mean duration of approximately 13 weeks. Studies with less than 30 subjects and ongoing blinded studies were excluded. The study databases were searched for possibly suicide-related adverse events (PSRAEs) that occurred during the double-blind phase of treatment, within 1 day of stopping randomized treatment, or on the first day of a protocol-specified tapering period. Adverse events (AEs) occurring prior to randomization or more than 1 day after discontinuation from randomized treatment were excluded. Only subjects taking DVPX or placebo were analyzed; subjects from comparator arms were excluded.

**Table 1:** Study descriptions and number of subjects

Indication	Study and year of publication	Description	Treatment duration, weeks	Dose and/or target trough drug level	Treatment group		Total, N = 2,319
						
					DVPX, N = 1,327	Placebo, N = 992	
Placebo-controlled studies							

Epilepsy	Willmore *et al*. 1996 [30]	Adjunctive therapy in CPS	16	90 mg/kg/day max	77	70	147
	
	Total				77 (6%)	70 (7%)	147 (6%)

Psychiatric	Pope *et al*. 1991 [31]	Acute mania^a^	3	50-100 μg/ml	20	23	43
	
	Bowden *et al*. 1994 [32]	Acute mania^a^	3	150 μg/ml	69	74	143
	
	Bowden *et al*. 2000 [33]	Mania maintenance	52	71-125 μg/ml	187	94	281
	
	Sachs *et al*. 2001 [34]	Bipolar depression	16	250 mg/day initial with titration^b^	23	22	45
	
	Hirschfeld *et al*. 2010 [35]	Acute mania^a^	3	20 mg/kg/day with increases allowed	146	78	224
	
	Tariot *et al*. 2001 [36]	Dementia^a^	6	30 mg/kg/day max	87	85	172
	
	Bowden *et al*. 2006 [37]	Acute mania^a^	3	85-125 μg/ml	192	185	377
	
	Hollander *et al*. 2003 [38]	Impulsive aggression	12	80-120 μg/ml, 30 mg/kg/day max	124	122	246
	
	Placebo-controlled study^c^	Dementia^a^	6	500 or 1,000 mg/day	78	43	121
	
	Total				926 (70%)	726 (73%)	1652 (71%)

Migraine	Mathew *et al*. 1995 [39]	Migraine prophylaxis	12	70-120 μg/ml	70	37	107
	
	Klapper 1997 [40]	Migraine prophylaxis	12	500, 1,000 or 1,500 mg/day	132	44	176
	
	Freitag *et al*. 2002 [41]	Migraine prophylaxis	12	500 or 1,000 mg/day	122	115	237
	
	Total				324 (24%)	196 (20%)	520 (22%)

High-dose DVPX vs low-dose DVPX							

				Trough levels	High-dose DVPX	Low-dose DVPX	

Epilepsy	Beydoun *et al*. 1997 [42]	Monotherapy in CPS	24	25-50 μg/ml and 80-150 μg/ml	131	134	265

^a^Inpatient study; all others were conducted in an outpatient setting; ^b^initiated at 250 mg, titrated by 250 mg/day on alternate days until reaching one of three criteria: serum trough concentration ≥45 μg/ml and HAM-D improvement ≥60% from baseline, ≥75 μg/ml and HAM-D ≥50% from baseline, or ≥95 μg/ml and HAM-D ≥30% from baseline; cData obtained from Abbott Protocol M99-082 clinical study report (unpublished). CPS = complex partial seizures; DVPX = divalproex sodium; HAM-D = Hamilton Rating Scale for Depression.

Deaths, serious adverse events and accidental injury events were identified. Preferred terms, verbatim terms, and comment fields in the study databases were searched to identify PSRAEs using the following text strings: 'suic', 'overdos', 'accident-', 'injur-', 'attempt', 'cut', 'gas', 'hang', 'hung', 'jump', 'mutilat-', 'self damag-', 'self harm', 'self inflict', 'self injur-', 'shoot', 'slash', 'poison', 'asphyxiation', 'suffocation', 'firearm', 'burn', 'drown', 'gun', 'immolate', and 'monoxide'. Additional information was obtained from clinical research forms, hospital records, consult results, and psychiatric rating scales.

Narrative summaries were generated for subjects identified with a PSRAE using a systematic approach as outlined in the Columbia Classification Algorithm for Suicide Assessment (C-CASA) [3]. Briefly, the C-CASA is a rating system designed to independently and reliably identify suicide-related adverse events from blinded narratives describing PSRAEs. In this evaluation, details in the narratives such as subject identifiers, sponsor name, investigator or site information, study drug, and concomitant medications were concealed to reduce potential bias during assessment. The blinded narratives were forwarded to a third party subject matter expert at Columbia University (New York, NY, USA) for severity rating of the PSRAEs using C-CASA methodology and definitions as described in Table 2[3]. Codes 7 or 8 were later recoded to 0. Only PSRAEs coded 1 to 4 were considered suicidality events and included in the FDA analysis.

**Table 2:** Suicidality event rating definitions

**Code no**.	Category	**C-CASA definition**^**a**^
1	Completed suicide	A self-injurious behavior that resulted in fatality and was associated with at least some intent to die as a result of the act

2	Suicide attempt	A potentially self-injurious behavior, associated with at least some intent to die, as a result of the act. Evidence that the individual intended to kill him/herself, at least to some degree, can be explicit or inferred from the behavior or circumstance. A suicide attempt may or may not result in actual injury.

3	Preparatory acts toward imminent suicidal behavior	The individual takes steps to injure him or herself, but is stopped by self or others from starting the self-injurious act before the potential for harm has begun

4	Suicidal ideation: passive, active, active with plans, type unknown	Passive thoughts about wanting to be dead or active thoughts about killing oneself, not accompanied by preparatory behavior

5	Self-injurious behavior, intent unknown	Self-injurious behavior where associated intent to die is unknown and cannot be inferred. The injury or potential for injury is clear, but why the individual engaged in that behavior is unclear.

6	Not enough information: death	Insufficient information to determine whether the event involved deliberate suicidal behavior or ideation. There is reason to suspect the possibility of suicidality but not enough to be confident that the event was not something other, such as an accident or psychiatric symptom.

7	Self-injurious behavior, no suicidal intent	Self-injurious behavior associated with no intent to die. The behavior is intended purely for other reasons, either to relieve distress (often referred to as 'self-mutilation', for example superficial cuts or scratches, hitting/banging, or burns) or to effect change in others or the environment.

8	Other: accident, death, psychiatric, medical	No evidence of any suicidality or deliberate self-injurious behavior associated with the event. The event is characterized as an accidental injury, psychiatric or behavioral symptoms only, or medical symptoms or procedure only.

9	Not enough information: non-death	Same as no. 6 above, with the event not resulting in death

^a^Definitions from [3]: Posner *et al*., *Am J Psych *2007, **164**:1035-1043.

C-CASA = Columbia Classification Algorithm for Suicide Assessment

### Statistical analysis methods

The meta-analysis methodology employed by the FDA in the evaluation of suicidality risk across 11 AEDs was utilized as the primary method to assess the risk of suicidality across multiple DVPX studies [4]. Delayed-release and extended-release DVPX formulations were combined and analyzed as DVPX treatment. To be consistent with the conservative approach employed by the FDA, the most severe suicidality event was included in the evaluation in situations where subjects experienced more than one event. The overall ORs of suicidality events across studies and associated 95% CIs were calculated using the exact method controlling for study [18]. For studies with no suicidality events, OR could not be calculated due to zeros in both the numerator and denominator. Therefore these studies could not be included in any of the overall OR analyses controlling for study. Zelen's test, an exact test for homogeneity of OR among studies, was conducted [18]. As a sensitivity analysis, SAS procedure GLIMMIX [19] (SAS, Cary, NC, USA) was used to estimate the OR using a generalized linear mixed model where study was considered as a random factor. The Mantel-Haenszel risk difference controlling for study and associated CI [20] were generated which included the zero-event studies. Relative risk analysis employing the exact method was also conducted [18]. This analysis used subject time as the unit of analysis rather than using the subject as the unit in the estimation of the OR. The overall absolute risks and relative risks from the pooled dataset were calculated for all placebo-controlled studies combined, for all placebo-controlled and low-dose-controlled studies combined, and by indication. These calculations did not use study as a stratification factor.

## Results

### Demographics, baseline characteristics, and duration

There were 13 placebo-controlled studies and 1 study comparing a high-dose with a low-dose of DVPX. Descriptions of the studies and corresponding numbers of subjects included in the analyses are presented in Table 2. All studies were conducted in the US and were completed prior to 2005.

Of 2,319 subjects from placebo-controlled studies (n = 1,327 for DVPX and n = 992 for placebo), 6% participated in an epilepsy study, 71% in psychiatry studies, and 22% in migraine studies. Subject demographic characteristics from these 13 studies are presented in Table 3. The mean age in both treatment groups was 44 years (range 9 to 100), and the majority of the subjects (83%) were Caucasian. The mean participation duration was 68 days in DVPX-treated subjects (range 1 to 400) and 57 days in placebo-treated subjects (1 to 391). There were no statistically significant differences between the two treatment groups in terms of age, gender, race, or duration of study participation.

**Table 3:** Demographic characteristics: placebo-controlled studies

Characteristic		Treatment group		Total, N = 2,319, n (%)	*P *value
				
		DVPX, N = 1,327, n (%)	Placebo, N = 992, n (%)		
Age, years	Mean ± SD	44 ± 18	44 ± 19		
	
	Least-squares mean	45	46		0.7454^a^
	
	Range	10 to 100	9 to 99		
	
	5 to 17	15 (1)	12 (1)	27 (1)	0.1413^b^
		
	18 to 24	131 (10)	83 (8)	214 (9)	
		
	25 to 30	140 (11)	138 (14)	278 (12)	
		
	31 to 64	855 (64)	617 (62)	1,472 (63)	
		
	≥65	186 (14)	142 (14)	328 (14)	

Gender	Female	740 (56)	544 (55)	1,284 (55)	0.3087^b^
		
	Male	587 (44)	448 (45)	1,035 (45)	

Race	White Caucasian	1,109 (84)	825 (83)	1,934 (83)	0.4430^b^
		
	Other	218 (16)	167 (17)	385 (17)	

Participation duration, days	Mean ± SD	68 ± 84	57 ± 64		0.2344^a^
		
	Least-squares mean	60	57		
		
	Range	1 to 400	1 to 391		

^a^*P *value for the treatment group difference is from a two-way analysis of variance with the terms of treatment and study.

^b^*P *value for the treatment group difference is from the Cochran-Mantel-Haenszel general association test controlling for study.

DVPX = divalproex.

### Suicidality events

When counting the single most severe suicidality event (codes 1-4) for subjects experiencing any event, 20 subjects from 5 of the 13 placebo-controlled studies experienced a suicidality event: 0 completed suicides, 4 suicide attempts (2 DVPX, 2 placebo), 1 preparatory act toward suicide (placebo), and 15 suicidal ideation (9 DVPX, 6 placebo). One low-dose DVPX subject in the epilepsy, adjunctive complex partial seizures (CPS) trial experienced suicidal ideation. No suicidality events occurred in the migraine prophylaxis or dementia studies. All but one of the subjects with suicidality events experienced the event in an outpatient setting, and 90% of the subjects who had suicidality events were white. Additional details regarding suicide-related events by severity and indication are presented in Table 4. The largest number of subjects experienced suicidality events during the 52-week mania maintenance study, seven (3.7%) in the DVPX group and seven (7.5%) in the placebo group. Three of the four suicide attempts occurred in this long-term bipolar maintenance study (two DVPX, one placebo), with the other occurring during a bipolar depression study (placebo). Three of the four subjects with suicide attempts had a known history of previous suicide attempts, and two of them had attempted suicide in the 12 months preceding study entry.

**Table 4:** Characteristics of suicidality events

Study	Treatment	Event, study day(s)	Age, years	Gender	Adverse event term(s)	Relevant history
Completed suicide: 0						

Suicide attempts: 4						

Mania maintenance	DVPX	241	21	Female	Overdose, suicide attempt	Benzodiazepine overdose after a family conflict. Family history of bipolar disorder; history of previous SA.

Mania maintenance	DVPX	313	43	Female	Manic depressive reaction, overdose, suicide attempt	Benzodiazepine overdose. Family history of alcoholism, ADHD; previous SA approximately 12 months prior; SADS-C suicidal tendency score was 1 (not at all) on day 308.

Mania maintenance	Placebo	71	29	Female	Overdose	Benzodiazepine + alcohol combination 'due to poor judgment'. Marital break-up and lost custody of children. Family history of anxiety, depression.

Bipolar depression^a^	Placebo	19	18	Male	Euphoria, abdominal pain, overdose	Amphetamine overdose. One SA in past year; mother died in past year; HAM-D rated as 0 (absent) and BPRS rated as 0 (not present) on day 15.

Preparatory acts toward imminent suicidal behavior: 1						

Mania maintenance	Placebo	29	26	Female	Depression	Severe depression and suicidal ideation 1 day post treatment. Family history of mood, eating, and drug abuse disorders; history of borderline personality disorder; five SA since 1983 (last approximately 1 year prior); SADS-C rated as 1 (not at all) on day -1, 2 (slight) on day 7 and 5 (severe) on day 29.

Suicidal ideation: 15^b^						

Epilepsy, adjunctive CPS	DVPX	19	21	Female	Depression	Severe thoughts of suicide that resolved the next day. History of drug abuse (approximately 1 year recovered), violent during seizures, and decreased mental sharpness; CBZ.

Acute mania	DVPX	2	33	Female	Depression	Moderate suicidal thoughts lasting 6 hours after a 'family event'. SADS-C rated as 0 (not at all) on day -1 and day 5.

Mania maintenance	DVPX	24	52	Male	Depression	Moderate suicidal ideation. Family history of suicide, abuse, electroconvulsive therapy, and residing in mental institution; history of obesity, diabetes, cardiovascular disease; SADS-C rated as 2 (slight) on day 15 and 4 (moderate) on day 30.

Mania maintenance	DVPX	23	31	Female	Depression	Severe depression, BDI indicated suicidal ideation. Family history of mood swings; SADS-C rated as 1 (not at all) on days -1 and 7, and 5 (severe) on day 24.

Mania maintenance	DVPX	196	22	Male	Depression	Severe depression and suicidal ideation. SADS-C rated as 3 (mild) on day 1.

Mania maintenance	DVPX	1	28	Male	Thinking abnormal thoughts	Moderate fleeting thoughts of wanting to hurt self (non-suicidal). SADS-C rated as 1 (not at all) on day -1, and 2 (slight) on day 18.

Mania maintenance	DVPX	44, 234	45	Male	Depression	Two episodes of suicidal ideation (severe and mild, respectively). Family history of depression; SADS-C rated as 6 (extreme) on day 55 and 3 on day 218; paroxetine.

Impulsive aggression	DVPX	16	37	Male	Depression	Severe hostility, depression and suicidal ideation, 'stress due to friend's death'. History of major depression, HIV positive (3 months), childhood physical abuse, possible PTSD; HAM-D rated as 1 (life not worth living) on day -22.

Impulsive aggression	DVPX	38	36	Female	Depression	Moderate feelings of worthlessness and hopelessness, mild thoughts of suicide. History of intermittent explosive disorder, alcohol dependence, cluster B personality disorder-borderline; HAM-D was rated as 0 (absent) on days 28 and 48.

Mania maintenance	Placebo	54	31	Male	Depression	Severe suicidal ideation and intent. Family history of depression; history of panic attacks; SADS-C rated as 3 (mild) on day 43 and 6 (extreme) on day 54; thyrosin, sertraline.

Mania maintenance	Placebo	188	45	Female	Manic depressive reaction	Severe suicidal ideation. Family history of bipolar disorder, depression; SADS-C rated as 1 (not at all) on day 175 and 5 (severe) on day 188.

Mania maintenance	Placebo	116	41	Female	Manic depressive reaction	Severe suicidal ideation and psychosis; SADS-C rated as 1 (not at all) on day 111 and 4 (moderate) on day 116.

Mania maintenance	Placebo	131	38	Male	Depression	Mild and transient suicidal ideation after ethanol consumption. Family history of bipolar disorder; SADS-C rated as 2 (slight) on day 115.

Mania maintenance	Placebo	39	32	Female	Psychotic depression	Severe depression and suicidal ideation. Family history of depression; SADS-C rated as 3 (mild) on day 28.

Impulsive aggression	Placebo	20	31	Female	Depression	Mild suicidal ideation with no plans/means that resolved within 2 hours. History of major depression, physical abuse, witnessed domestic violence, borderline cluster B personality disorder; HAM-D rated as 0 (absent) on day -15 and 28.

Epilepsy, monotherapy	Low-dose DVPX	17	33	Female	Depression	Moderate depression and thoughts of suicide. Marital and financial issues; history of anxiety, CBZ.

^a^This subject also experienced an event of suicidal ideation (code 4) on day 17; only the most severe event is included in this table.

^b^N = 15 in placebo-controlled studies when counting the most severe event in patients experiencing >1 event.

ADHD = attention-deficit hyperactivity disorder; BDI = Beck Depression Inventory; BPRS = Brief Psychiatric Rating Scale suicidal thoughts; CBZ = carbamazepine; CPS = complex partial seizures; DVPX = divalproex sodium; HAM-D = Hamilton Depression Rating Scale suicide score; PTSD = post-traumatic-stress disorder; SA = suicide attempt; SADS-C = Schedule for Affective Disorders and Schizophrenia-Change Version suicidal tendency score.

Two subjects reported more than one suicidality event during a study. One subject taking DVPX experienced suicidal ideation on days 44 and 234 of the bipolar maintenance study. Another subject taking placebo in the bipolar depression study experienced suicidal ideation on day 17, and attempted suicide on day 19. This subject had previously attempted suicide within the 12 months prior to entering the study.

### Meta-analysis results

The incidence of suicidality events, ORs by study, and estimated overall OR across five placebo-controlled studies with at least one event are presented in Table 5. None of the ORs comparing DVPX with placebo were statistically significantly different from 1. A total of 11 (0.83%) subjects exposed to DVPX experienced suicidal behavior or ideation while 9 (0.91%) placebo-treated subjects reported suicidality events. The overall estimated OR of suicidal behavior or ideation was 0.72 (95% CI 0.29 to 1.84). For the placebo-controlled studies, the OR for suicidal behavior was 0.37 (95% CI 0.04 to 2.58), and the OR for suicidal ideation was 0.90 (95% CI 0.31 to 2.79).

**Table 5:** Odds ratios by study and overall odds ratio estimated for low-dose and placebo-controlled studies

Study	Population	Treatment group		OR (95% CI)
			
		**DVPX, N = 1,327, n/N**^**a**^	**Placebo, N = 992, n/N**^**a**^	
Placebo-controlled studies				

Willmore *et al*. 1996 [30]	Epilepsy	1/77	0/70	2.76 (0.11 to 68.98)

Pope *et al*. 1991 [31]	Acute mania	0/20	0/23	-

Bowden *et al*. 1994 [32]	Acute mania	0/69	0/74	-

Bowden *et al*. 2000 [33]	Mania maintenance	7/187	7/94	0.48 (0.16 to 1.42)

Sachs *et al*. 2001 [34]	Bipolar depression	0/23	1/22	0.31 (0.01 to 7.89)

Hirschfeld *et al*. 2010 [35]	Acute mania	1/146	0/78	1.62 (0.07 to 40.20)

Tariot *et al*. 2001 [36]	Dementia	0/87	0/85	-

Bowden *et al*. 2006 [37]	Acute mania	0/192	0/185	-

Hollander *et al*. 2003 [38]	Impulsive aggression	2/124	1/122	1.98 (0.18 to 22.16)

Placebo-controlled study^b^	Dementia	0/78	0/43	-

Mathew *et al*. 1995 [39]	Migraine	0/70	0/37	-

Klapper 1997 [40]	Migraine	0/132	0/44	-

Freitag *et al*. 2002 [41]	Migraine	0/122	0/115	-

Overall (placebo-controlled)		11/557^c^	9/386^c^	0.72 (0.29 to 1.84)

High-dose DVPX vs low-dose DVPX				

		High-dose DVPX	Low-dose DVPX	

Beydoun *et al*. 1997 [42]	Epilepsy	0/131	1/134	0.34 (0.01 to 8.38)

Overall (all studies)		11/688^c^	10/520^c^	0.66 (0.27 to 1.64)

^a^N = the number of subjects treated in the study for the prospective treatment group.

^b^Data obtained from Abbott Protocol M99-082 clinical study report (unpublished).

^c^The denominator presents the total number of subjects in the studies with at least one suicidality event and therefore included in the overall OR calculation.

DVPX = divalproex sodium; NA = not applicable; OR = odds ratio; - = zero-event studies, OR cannot be estimated.

Zelen's test for the null hypothesis that all studies had a common OR for suicidality events had a *P *value of 0.467, indicating a homogenous OR of suicidality events across studies. The OR estimated from a generalized linear mixed model with fixed effect for treatment and a random effect for study was 0.74 (95% CI 0.30 to 1.82). The overall risk difference between DVPX treatment and the placebo group was -2.75 (95% CI -10.68 to 5.17) per 1,000 subjects and was not significantly different from zero. The relative risk comparing DVPX with placebo, adjusting for subject exposure, was 0.64 (95% CI 0.26 to 1.62) and not statistically significantly different from 1. The statistical inferences from these sensitivity analyses are consistent with those obtained from the primary analysis.

The single low-dose-controlled study was not included in the previous analyses because the design did not include a placebo control. In this monotherapy study in subjects with complex partial seizures, one subject in the low-dose DVPX treatment group experienced suicidal ideation. When analyzed individually, the OR of suicidal behavior or ideation in this study between the high-dose group and the low-dose group was 0.34 (95% CI 0.01 to 8.38). Combining the low-dose study with the placebo-controlled studies and pooling the low-dose group with the placebo group yielded an OR of 0.66 (95% CI 0.27 to 1.64) for suicidality events (Table 5).

Table 6 presents the overall absolute risk, relative risk, and risk difference for all placebo-controlled and low-dose-controlled studies (total and by indication), as well as for all placebo-controlled studies combined by pooling the subjects from respective studies together. The relative risk was numerically higher in the epilepsy group (0.98) than in the psychiatric group (0.87).

**Table 6:** Absolute and relative risk by indication and overall (pooled datasets)

	Placebo		DVPX		Relative risk	Risk difference per 1,000 subjects
	
	n/N	Absolute risk per 1,000 subjects	n/N	Absolute risk per 1,000 subjects	Incidence of events in DVPX subjects/incidence in placebo subjects	Additional DVPX subjects with events
Placebo-controlled and low-dose-controlled studies						

Indication:						

Epilepsy	1/204	4.90	1/208	4.81	0.98	-0.09

Psychiatric	9/726	12.40	10/926	10.80	0.87	-1.60

Migraine	0/196	0.00	0/324	0.00	-	0.00

Total	10/1,126	8.88	11/1,458	7.54	0.85	-1.34

Placebo-controlled studies						

Total	9/992	9.07	11/1,137	8.29	0.91	-0.78

DVPX = divalproex sodium; n = number of subjects with events across studies; N = number of subjects treated across studies; - = relative risk cannot be calculated due to zero events in studies.

## Discussion

Higher rates of suicidality have been previously reported for populations that take AEDs for different indications, particularly subjects with epilepsy [10] and bipolar disorder [2,10]. Determining whether AED use increases suicidality in patients taking these medications is a complex task, and research in this area continues. One recent example is a large pharmacoepidemiological study of patients taking AEDs or lithium as monotherapy for bipolar disorder conducted by Gibbons *et al*. [14]. Medical claims data from 47,918 patients with bipolar disorder were studied. Patient data was included if it encompassed at least 1 year pre-illness and post-illness index date. The authors reported that suicide attempt rates were significantly greater before AED therapy was initiated (72 per 1,000 person-years) compared to 13 per 1,000 person-years after treatment began (*P *< 0.001). For patients not treated with any central nervous system drug, suicide attempts were 15 per 1,000 person-years, compared with 3 per 1,000 person-years in patients treated with an AED (*P *< 0.001) [14].

In the present analysis, treatment with DVPX in a variety of conditions did not appear to increase the risk of suicide-related AEs relative to that of placebo, consistent with the DVPX OR of 0.72 (95% CI 0.29 to 1.84) estimated by the FDA during individual AED analyses. When examining all 14 DVPX studies combined, the relative risk was <1 for both the epilepsy and psychiatric populations, but slightly higher in epilepsy subjects compared to psychiatric study subjects. These estimates correspond to the FDA results indicating that the relative risk for suicidal thoughts or behavior was higher in clinical trials for epilepsy than in clinical trials for psychiatric or other conditions.

Although systematic retrospective reviews of clinical study data yield useful information, this approach has some limitations. The observation that treatment with DVPX did not increase the risk of suicidality events differs from the overall conclusions of FDA meta-analysis of 11 AEDs. This inconsistency may be related to the size of the datasets analyzed. The DVPX data are obviously a subset of the much larger dataset collected by the FDA, and this smaller population may have prevented the detection of uncommon events. Additionally, the AEDs analyzed belong to multiple pharmacological classes. This may be an important factor to consider when interpreting pooled data in the determination of suicidality risk associated with the use of these drugs because the different mechanisms of action could be a confounding factor. None of the DVPX clinical studies were specifically designed to assess suicidality *a priori*, and the study designs, DVPX doses, and types of data collected from a variety of populations was highly variable. The retrospective nature of the analysis may have led to ascertainment bias, making it difficult to draw definitive conclusions regarding the causality of events. In addition, data from controlled trials may not translate to larger populations. Not only were subjects selected based on specific study criteria, but it is also possible that protocol-specified interventions may have alleviated participants' psychiatric symptoms. The larger number of events reported in the 52-week bipolar maintenance study may have been associated with the longer duration of follow-up or the illness *per se*. As 83% of study participants in the pooled dataset were Caucasian, racial or regional differences in suicide-related adverse events may not be reflected in this dataset.

It is important to note that when interpreting research regarding suicidality it is not possible to predict whether an event of lesser severity such as suicidal ideation will ultimately lead to suicide [21-23]. It is clear that evidence of suicidality must be clinically assessed to prevent progression to more serious events. A recent analysis of the National Comorbidity Survey Replication indicated that in a population of community dwelling US adults, approximately 80% of those who attempt suicide had a psychiatric disturbance prior to the suicide attempt [23]. Major depression, affective disorders, psychoses, substance abuse, and personality disorders have been reported to place patients with epilepsy at higher risk of suicide. In a prospective analysis of a large group of bipolar patients followed for 2 years, history of suicide attempt (OR = 4.52, *P *< 0.0001) and the percentage of days depressed during the previous year (OR = 1.16, *P *= 0.036) were significantly related to suicide attempts and completions [24]. Increasing awareness of suicidality, assessing prior suicide-related events, and utilizing appropriate psychiatric screening measures may therefore serve to minimize risks of suicidality [15,24-29]. The employment of prospective monitoring to assess suicidal ideation and behaviors over time should overcome the limited nature of retrospective evaluations while enhancing patient safety and investigative outcomes.

## Conclusions

In this meta-analysis, divalproex sodium does not appear to increase the risk of suicide-related adverse events relative to placebo. Screening assessments combined with vigilance on the part of clinicians remain important strategies for reducing suicidality in patients treated with AEDs.

## Competing interests

This study was supported by Abbott. Abbott personnel performed statistical analyses on the data. All authors are employees of Abbott, receive salary and other compensation from Abbott, and hold Abbott stock options and/or stock. In addition, KT and MS are Abbott patent holders.

## Authors' contributions

All authors participated in the study design, the coordination of the study, and participated in drafting the manuscript. In addition, YP and WR performed the statistical analysis. All authors read and approved the final manuscript.
